# Predictors of textbook outcome following oesophagogastric cancer surgery

**DOI:** 10.1093/dote/doae023

**Published:** 2024-03-24

**Authors:** Ganesh K Velayudham, Alexander Dermanis, Sivesh K Kamarajah, Ewen A Griffiths

**Affiliations:** College of Medical and Dental Sciences, University of Birmingham, Birmingham, UK; Department of Upper Gastrointestinal Surgery, University Hospitals Birmingham NHS Foundation Trust, Birmingham, UK; Department of Upper Gastrointestinal Surgery, University Hospitals Birmingham NHS Foundation Trust, Birmingham, UK; Institute of Applied Health Research, University of Birmingham, Birmingham, UK; Department of Upper Gastrointestinal Surgery, University Hospitals Birmingham NHS Foundation Trust, Birmingham, UK; Institute of Immunology and Immunotherapy, College of Medical and Dental Sciences, University of Birmingham, Birmingham, UK

**Keywords:** gastrectomy, oesophagectomy, oesophagogastric cancer, quality improvement, textbook outcome

## Abstract

Textbook outcome (TO) is a composite measure representing an ideal perioperative course, which has been utilized to assess the quality of care in oesophagogastric cancer (OGC) surgery. We aim to determine TO rates among OGC patients in a UK tertiary center, investigate predictors of TO attainment, and evaluate the relationship between TO and survival. A retrospective analysis of a prospectively collected departmental database between 2006 and 2021 was conducted. Patients that underwent radical OGC surgery with curative intent were included. TO attainment required margin-negative resection, adequate lymphadenectomy, uncomplicated postoperative course, and no hospital readmission. Predictors of TO were investigated using multivariable logistic regression. The association between TO and survival was evaluated using Kaplan–Meier analysis and Cox regression modeling. In sum, 667 esophageal cancer and 312 gastric cancer patients were included. TO was achieved in 35.1% of esophagectomy patients and 51.3% of gastrectomy patients. Several factors were independently associated with a low likelihood of TO attainment: T3 stage (odds ratio (OR): 0.41, 95% confidence interval (CI) [0.22–0.79], *p* = 0.008) and T4 stage (OR:0.26, 95% CI [0.08–0.72], *p* = 0.013) in the esophagectomy cohort and high BMI (OR:0.93, 95% CI [0.88–0.98], *p* = 0.011) in the gastrectomy cohort. TO attainment was associated with greater overall survival and recurrence-free survival in esophagectomy and gastrectomy cohorts. TO is a relevant quality metric that can be utilized to compare surgical performance between centers and investigate patients at risk of TO failure. Enhancement of preoperative care measures can improve TO rates and, subsequently, long-term survival.

## INTRODUCTION

Evaluating surgical outcomes is necessary for assessing hospital performance, identifying factors influencing healthcare quality, and empowering patient decision-making.^1^ Metrics including surgical mortality and complications rates have been utilized to measure the quality of surgical care.^2^ These single outcomes have low event rates, limiting statistical power, and do not describe the entire surgical process.^3–5^ The concept of a textbook outcome (TO) addresses these limitations, representing a composite measure encompassing intraoperative and postoperative indicators that collectively define an optimal perioperative course.^6^ Originally developed for colorectal surgery, it has been adapted for oesophagogastric cancer (OGC) procedures by the Dutch Upper Gastrointestinal Cancer Audit (DUCA) group.^3^

Subsequent studies adopting this definition have identified predictive clinicopathologic factors of TO attainment and demonstrated its association with long-term survival.^7–10^ Whereas TO in OGC patients has been investigated by several international institutions, there is a necessity to evaluate this concept in a high-volume UK center. Investigation of TO rates would enable the evaluation of perioperative care at our institution and comparison with other specialist centers. This study primarily aims to determine TO rates in OGC patients within a UK tertiary center. Secondary objectives include identifying predictors of TO and assessing the relationship between TO attainment and survival.

## METHODS

### Study design and setting

A single-center, retrospective, observational study was conducted of patients undergoing surgical treatment for OG cancer at the Queen Elizabeth Hospital Birmingham (QEHB), a high-volume, tertiary center. The hospital receives referrals from a regional catchment area of approximately 3,240,000 people. Operative approach was decided by the individual surgeons involved and the MDT but included review of all staging investigations and intra-operative assessment in difficult cases (including frozen section if required). In type II gastroesophageal tumors, if the patient is fit for trans-thoracic access, the most common operation was a trans-thoracic esophagectomy. In selected cases, for example, where the patient is unfit for thoracotomy, an extended total gastrectomy was performed. A routine two-field lymphadenectomy was performed for esophagectomy (thoracic and abdominal), and a D1.5 lymphadenectomy was undertaken in the abdominal phase and gastrectomy procedures (i.e. lymphadenectomy along the common hepatic and splenic arteries, but routine splenectomy and distal pancreatectomy was avoided).

### Study population

Patients undergoing elective esophagectomy or gastrectomy with curative intent between 1st January 2006 and 31st December 2021 were included. Patients were excluded if they underwent (i) non-elective procedures, (ii) resections for benign indications, (iii) prophylactic gastrectomy, (iv) open and close procedure, (v) non-resectional procedures, (vi) surgery for non-epithelial OGC, (vii) simple wedge resections, and (viii) insufficient information regarding TO parameters.

### TO definition

TO was achieved when the following nine parameters were met: no intraoperative complication, margin-negative (R0) resection, 15 or more lymph nodes examined, no severe postoperative complication, no re-intervention within 30 days of procedure, no unplanned ICU admission, length of hospital stay ≤21 days, no 90-day postoperative mortality, and no unplanned hospital readmission within 30 days of discharge. Tumor margins were defined in accordance with Royal College of Pathologists guidance.^11^ Margin-negative tumors were defined as a > 1 mm proximal, distal, and circumferential margin for esophagectomy and > 1 mm proximal and distal margin for gastrectomy. R1 tumors (microscopic margin involvement) were subdivided as R1a (<1 mm margin involvement) and R1b (absolute margin involvement). The overall R status was determined by the resection margin (proximal, distal, or circumferential) with the smallest distance between tumor and margin. The severity of postoperative complications was graded according to the Clavien–Dindo classification.^12^ The original definition formulated by the DUCA group^3^ was modified in this study through the exclusion of the parameter ‘radical resection according to the surgeon at the end of surgery’ as this data was not available and is subjective. Furthermore, the definition of severe postoperative complications was amended to Clavien–Dindo III or above to reflect corresponding changes in recent studies.^2^^,^^13–16^ Additionally, the definition of postoperative mortality was defined to 90-day mortality in keeping with other TO papers highlighting that it is a more clinically relevant endpoint.^17^

### Data collection and outcomes

Records were identified from a prospectively collected database to gather data regarding patient demographics, tumor pathology, management, perioperative course, and survival. Patient, tumor, and treatment factors were investigated for association with TO attainment. The Charlson Co-morbidity Index is often considered the gold-standard measure to assess clinical comorbidity.^18^ Due to insufficient comorbidity data, this score could not be calculated, and a comorbidity count (total no. of comorbidities) was measured as an alternative. Pathological TNM classification was used to stage tumors.^19^ Survival data, including dates of death, recurrence, and final follow-up, were collected to investigate the relationships between TO attainment and overall survival (OS) and recurrence-free survival (RFS). Recurrence and mortality were considered endpoints in the calculation of RFS.

### Statistical analysis

Categorical data were represented by frequencies and percentages and assessed using the chi-squared test. Non-normally distributed continuous data were summarized with medians and inter-quartile ranges and compared using the Mann–Whitney U test. Predictors of TO were identified through univariable and multivariable logistic regression, and outcomes were reported as odds ratios (OR) with 95% confidence intervals (CI). Survival analysis employed Kaplan–Meier curves and the log-rank test. The Cox proportional hazards model adjusted for confounders, reporting adjusted hazard ratios (HR) with 95% CI. Significance was defined as *p* ≤ 0.05, and analyses were conducted using R version 4.2.3.

## RESULTS

**Fig. 1:**
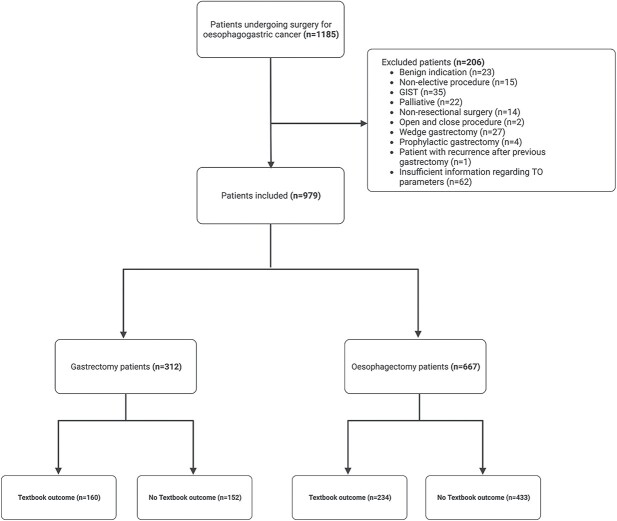
Consort diagram showing patients included in the study, GIST, Gastrointestinal stromal tumor.

Out of 1185 patients identified, a total of 312 gastric cancer patients and 667 esophageal cancer patients were included in this study (Fig. 1).

### Baseline characteristics

Table 1 reports the demographic and clinical data of the patients and the number of patients with missing data. Baseline characteristics were compared between TO and non-TO groups in esophagectomy and gastrectomy cohorts separately. Significant differences in comorbidity count, T stage, N stage, and anastomosis type were observed in the esophageal cancer group. BMI and surgical approach were significantly different in the gastric cancer group (Table 2).

**Table 1:** Patient demographics

	**Esophageal cancer**		**Gastric cancer**	
	**Patients† (*n*)**	**Statistic**	**Patients† (*n*)**	**Statistic**
**Patient characteristics**				
**Age** (median, IQR)	667	66.0 (59.0–72.0)	312	71.0 (60.0–77.0)
**Gender**, *n* (%)	667		312	
Female		135 (20.2)		96 (30.8)
Male		532 (79.8)		216 (69.2)
**BMI** (median, IQR)	655	26.5 (23.9–30.1)	306	26.3 (23.1–29.5)
**ASA Grade**, *n* (%)	661		312	
1		93 (14.1)		31 (9.9)
2		395 (59.8)		170 (54.5)
3		165 (25.0)		105 (33.7)
4		8 (1.2)		5 (1.6)
Missing		6 (0.9)		1 (0.3)
**Comorbidity count**, *n* (%)	667		312	
0		248 (37.2)		90 (28.8)
1		214 (32.1)		91 (29.2)
>2		205 (30.7)		131 (42.0)
Tumor **characteristics**				
Tumor **histology, *n* (%)**	667		312	
Adenocarcinoma		549 (82.3)		307 (98.4)
SCC		99 (14.8)		0 (0)
Other		19 (2.8)		5 (1.6)
**T stage, *n* (%)**	667		312	
T0		54 (8.1)		12 (3.80
T1		112 (16.8)		72 (23.1)
T2		82 (12.3)		54 (17.3)
T3		382 (57.3)		100 (32.1)
T4		37 (5.5)		74 (23.7)
**N Stage, *n* (%)**	667		312	
N0		296 (44.4)		147 (47.1)
N1		224 (33.6)		58 (18.6)
N2		90 (13.5)		57 (18.3)
N3		57 (8.5)		50 (16.0)
**M Stage, *n* (%)**	667		312	
M0		658 (98.7)		303 (97.1)
M1		9 (1.3)		9 (2.9)
**Treatment characteristics**				
**Neoadjuvant therapy, *n* (%)**	667		312	
No		142 (21.3)		170 (54.5)
Yes		525 (78.7)		142 (45.5)
**Type of resection (esophectomy)**	667		NA	
Transthoracic—Ivor Lewis		621 (93.1)		
Transthoracic—McKeown		44 (6.6)		
Transhiatal		2 (0.3)		
**Type of resection (gastrectomy)**	NA		312	
Total				142 (45.5)
Subtotal				170 (54.5)
**Surgical approach (esophagectomy), *n* (%)**	667		NA	
Open		148 (22.2)		
Hybrid		385 (57.7)		
Total minimally invasive		134 (20.1)		
**Surgical approach (gastrectomy), *n* (%)**	NA		312	
Open				286 (91.7)
Laparoscopic				26 (8.3)
**Anastomosis type, *n* (%)**	643		282	
Circular stapled		371 (57.7)		95 (33.7)
Hand sewn		145 (22.6)		85 (30.1)
Linear stapled		127 (19.8)		102 (36.2)
Missing		24 (3.7)		30 (10.6)

Data reported as median (IQR) or *n* (%), as applicable. † Number of patients for whom data were available.

BMI, body mass index; ASA, American Society of Anaesthesiologists; SCC, squamous cell carcinoma.

**Table 2:** Comparison of patient, tumor, and treatment characteristics between TO and non-TO groups in esophageal and gastric cancer cohorts

	**Esophageal cancer**				**Gastric cancer**			
	**Patients (*n*)**	**Non-TO (*n* = 433)**	**TO (*n* = 234)**	***p*-value**	**Patients (*n*)**	**Non-TO (*n* = 152)**	**TO (*n* = 160)**	***p*-value**
**Patient characteristics**								
**Age (median, IQR)**	667	66.0 (60.0–72.0)	65.0 (58.0–71.0)	0.120^**^	312	71.0 (60.8–78.0)	71.0 (60.0–76.0)	0.554^*^^*^
**Sex, *n* (%)**	667			0.166^*^	312			0.503^*^
Female		95 (70.4)	40 (29.6)			50 (52.1)	46 (47.9)	
Male		338 (63.5)	194 (36.5)			102 (47.2)	114 (52.8)	
**BMI (median, IQR)**	655	26.3 (23.9–30.2)	26.7 (24.2–29.6)	0.816^**^	306	27.1 (23.2–30.6)	25.7 (23.1–28.6)	**0.015** ^*^^*^
**ASA Grade, *n* (%)**	661			0.134^*^	312			0.564^*^
1		63 (67.7)	30 (32.3)			18 (58.1)	13 (41.9)	
2		242 (61.3)	153 (38.7)			78 (45.9)	92 (54.1)	
3		117 (70.9)	48 (29.1)			52 (49.5)	53 (50.5)	
4		7 (87.5)	1 (12.5)			3 (60.0)	2 (40.0)	
Missing		4 (66.7)	2 (33.3)			1 (100.0)	0 (0.0)	
**Comorbidity count, *n* (%)**	667			**0.027** ^*^	312			0.226^*^
0		145 (58.5)	103 (41.5)			42 (46.7)	48 (53.3)	
1		148 (69.2)	66 (30.8)			39 (42.9)	52 (57.1)	
>2		140 (68.3)	65 (31.7)			71 (54.2)	60 (45.8)	
Tumor **characteristics**								
Tumor **histology, *n* (%)**	667			0.160^*^	312			0.954^*^
Adenocarcinoma		348 (63.4)	201 (36.6)			149 (48.5)	158 (51.5)	
SCC		70 (70.7)	29 (29.3)			NA	NA	
Other		15 (78.9)	4 (21.1)			3 (60.0)	2 (40.0)	
**T Stage, *n* (%)**	667			**<0.001** ^*^	312			0.315^*^
T0		26 (48.1)	28 (51.9)			5 (41.7)	7 (58.3)	
T1		63 (56.2)	49 (43.8)			32 (44.4)	40 (55.6)	
T2		44 (53.7)	38 (46.3)			26 (48.1)	28 (51.9)	
T3		270 (70.7)	112 (29.3)			45 (45.0)	55 (55.0)	
T4		30 (81.1)	7 (18.9)			44 (59.5)	30 (40.5)	
**N Stage, *n* (%)**	667			**0.007** ^*^	312			0.382^*^
N0		175 (59.1)	121 (40.9)			69 (46.9)	78 (53.1)	
N1		147 (65.6)	77 (34.4)			27 (46.6)	31 (53.4)	
N2		66 (73.3)	24 (26.7)			26 (45.6)	31 (54.4)	
N3		45 (78.9)	12 (21.1)			30 (60.0)	20 (40.0)	
**M Stage, *n* (%)**	667			0.644^*^	312			0.938^*^
M0		426 (64.7)	232 (35.3)			147 (48.5)	156 (51.5)	
M1		7 (77.8)	2 (22.2)			5 (55.6)	4 (44.4)	
**Treatment characteristics**								
**Neoadjuvant therapy, *n* (%)**	667			0.646^*^	312			0.196^*^
No		95 (66.9)	47 (33.1)			89 (52.4)	81 (47.6)	
Yes		338 (64.4)	187 (35.6)			63 (44.4)	79 (55.6)	
**Surgical approach (esophagectomy), *n* (%)**	667			0.408^*^	NA			NA
Open		99 (66.9)	49 (33.1)					
Hybrid		242 (62.9)	143 (37.1)					
Total minimally invasive		92 (68.7)	42 (31.3)					
**Surgical approach (gastrectomy), *n* (%)**					312			**0.017** ^*^
Open						133 (46.5)	153 (53.5)	
Laparoscopic						19 (73.1)	7 (26.9)	
**Anastomosis type, *n* (%)**	643			**0.034** ^*^	282			0.239^*^
Circular stapled		244 (65.8)	127 (34.2)			45 (47.4)	50 (52.6)	
Hand sewn		98 (67.6)	47 (32.4)			44 (51.8)	41 (48.2)	
Linear stapled		71 (55.9)	56 (44.1)			44 (43.1)	58 (56.9)	
Missing		20 (83.3)	4 (16.7)			19 (63.3)	11 (36.7)	

^*^ *p*-value calculated using Chi-squared test.

^**^ *p*-value calculated using Mann–Whitney U test. Data have been presented using frequency (*n*), percentage (%) or median, interquartile range (IQR). Significant *p*-values (<0.05) are in bold.

BMI, body mass index; ASA, American Society of Anaesthesiologists; SCC, squamous cell carcinoma.

### TO rates

**Fig. 2:**
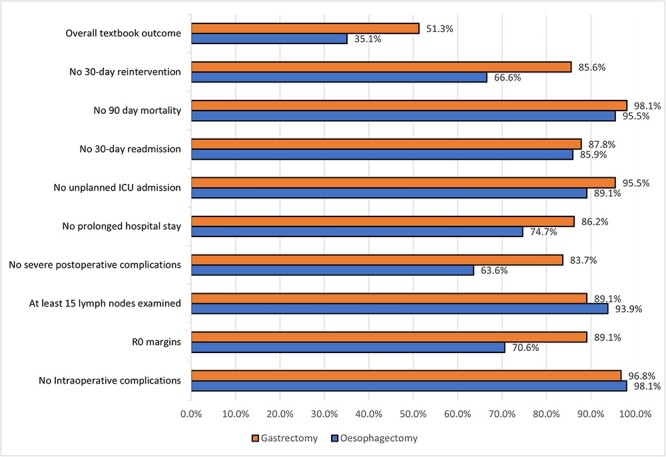
Bar chart comparing the rates of TO and its individual parameters between esophagectomy and gastrectomy patients. Abbreviations: ICU, intensive care unit; R0, margin-negative resection.

TO was achieved in 35.1 and 51.3% of esophagectomy and gastrectomy patients, respectively. The least achieved parameter in both groups was ‘no severe postoperative complications’ (Fig. 2).

### Predictors of TO

Multivariable logistic regression analysis identified predictive variables of TO attainment. In the esophageal cancer group, T3 (*p* = 0.008) and T4 (*p* = 0.013) tumor stages were predictive for non-TO attainment. In the gastric cancer cohort, high BMI (*p* = 0.011) was associated with a lower likelihood of TO (Table 3).

**Table 3:** Results of multivariable logistic regression analysis identifying patient, tumor, and treatment factors associated with TO attainment

	**OR (multivariable analysis)**	
	**Gastric cancer**	**Esophageal cancer**
**Patient characteristics**		
**Age**	1.00 (0.98–1.03, *p* = 0.725)	0.98 (0.96–1.00, *p* = 0.081)
**Sex**		
F	Ref-	Ref-
M	1.14 (0.66–1.95, *p* = 0.645)	1.27 (0.80–2.05, *p* = 0.312)
**BMI**	**0.93 (0.88–0.98, *p* = 0.011)**	0.98 (0.95–1.02, *p* = 0.360)
**ASA Grade**		
1	Ref-	Ref-
2	1.49 (0.59–3.80, *p* = 0.399)	1.31 (0.78–2.22, *p* = 0.312)
3	1.79 (0.67–4.93, *p* = 0.250)	1.07 (0.58–1.99, *p* = 0.834)
4	2.63 (0.24–25.97, *p* = 0.402)	0.31 (0.02–2.01, *p* = 0.298)
Missing	NA	2.13 (0.25–15.22, *p* = 0.450)
**Comorbidity count**		
0	Ref-	Ref-
1	1.34 (0.67–2.71, *p* = 0.407)	0.68 (0.44–1.04, *p* = 0.075)
>2	0.63 (0.31–1.26, *p* = 0.191)	0.74 (0.47–1.15, *p* = 0.185)
Tumor **characteristics**		
Tumor **histology**		
Adenocarcinoma	Ref-	Ref-
Other	0.67 (0.08–4.46, *p* = 0.677)	0.52 (0.14–1.55, *p* = 0.275)
SCC	NA	0.59 (0.34–1.01, *p* = 0.059)
**T stage**		
T0	Ref-	Ref-
T1	1.01 (0.25–3.87, *p* = 0.985)	0.73 (0.36–1.50, *p* = 0.399)
T2	0.82 (0.19–3.35, *p* = 0.788)	0.79 (0.38–1.64, *p* = 0.532)
T3	0.67 (0.16–2.60, *p* = 0.567)	**0.41 (0.22–0.79, *p* = 0.008)**
T4	0.33 (0.07–1.37, *p* = 0.132)	**0.26 (0.08–0.72, *p* = 0.013)**
**N Stage**		
N0	Ref-	Ref-
N1	1.39 (0.66–2.93, *p* = 0.388)	1.04 (0.68–1.57, *p* = 0.868)
N2	1.34 (0.63–2.92, *p* = 0.450)	0.79 (0.43–1.42, *p* = 0.433)
N3	0.81 (0.35–1.87, *p* = 0.620)	0.57 (0.26–1.17, *p* = 0.135)
**M Stage**		
M0	Ref-	Ref-
M1	0.78 (0.16–3.86, *p* = 0.758)	0.60 (0.08–2.76, *p* = 0.553)
**Treatment characteristics**		
**Neoadjuvant therapy**		
No	Ref-	Ref-
Yes	1.74 (0.99–3.08, *p* = 0.054)	1.21 (0.76–1.96, *p* = 0.429)
**Surgical approach (gastrectomy)**		
Open	Ref-	-
Laparoscopic	**0.35 (0.12–0.97, *p* = 0.050)**	-
**Surgical approach (esophagectomy)**		
Open	-	Ref-
Hybrid	-	1.08 (0.69–1.72, *p* = 0.729)
Total MIO	-	0.90 (0.53–1.54, *p* = 0.706)
**Anastomosis type**		
Circular stapled	Ref-	Ref-
Hand sewn	0.81 (0.43–1.53, *p* = 0.511)	0.98 (0.61–1.55, *p* = 0.927)
Linear stapled	1.68 (0.88–3.24, *p* = 0.121)	1.25 (0.78–1.99, *p* = 0.358)
Missing	0.71 (0.25–2.01, *p* = 0.523)	0.45 (0.12–1.29, *p* = 0.172)

OR reported with 95% CI and *p*-value. Significant results (*p* < 0.05) are in bold.

BMI, body mass index; ASA, American Society of Anaesthesiologists; SCC, squamous cell carcinoma; MIO, minimally invasive oesophagectomy.

### Survival outcomes

**Fig. 3:**
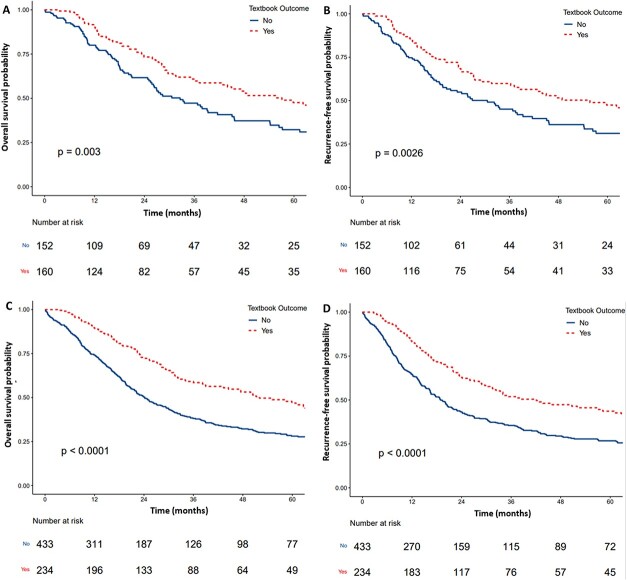
Kaplan–Meier curves comparing survival between TO and non-TO groups for **(A)** overall-survival in gastrectomy cohort, **(B)** RFS in gastrectomy cohort, **(C)** OS in esophagectomy cohort, **(D)** RFS in esophagectomy cohort. (**A**) *p* = 0.003, (**B**) *p* = 0.0026, (**C**) *p* < 0.0001, (**D**) *p* < 0.0001 (log-rank test).

The median follow-up in gastrectomy patients was 41.6 months. 3-year OS was 60.9% in patients with and 47.2% in patients without TO (*p* = 0.003) (Fig. 3A). RFS was 59.1% in patients with and 45.1% in those without a TO (*p* = 0.0026) (Fig. 3B). The proportional hazards model (Tables S1 and S2) demonstrated that TO was associated with greater OS (HR: 0.63, 95% CI [0.43–0.91]; *p* = 0.014) and RFS (HR: 0.64, 95% CI [0.44–0.91]; *p* = 0.013).

The median follow-up for esophagectomy patients was 30.7 months. The 3-year OS was 58.5% compared to 38.1% in those with and without TO (*p* < 0.0001) (Fig. 3C). The 3-year RFS was 51.9% in the TO group and 35.5% in the non-TO group (*p* < 0.0001) (Fig. 3D). In the multivariable Cox model (Tables S3 and S4), TO was found to be independently predictive of greater OS (HR: 0.60, 95% CI [0.48–0.76]; *p* < 0.001) and RFS (HR: 0.63, 95% CI [0.51–0.79]; *p* < 0.001).

## DISCUSSION

Overall, 35.1% of esophageal cancer patients achieved TO, which is concordant with studies reporting rates between 30 and 40%.^8–10^ Differences in neoadjuvant and surgical management contribute to variation in TO rates. Transthoracic esophagectomy predominated at our center, with only 0.3% of patients undergoing transhiatal esophagectomy, whereas in other centers, the transhiatal technique was more common.^7–9^ The morbidity rates are comparable; however, lymph node retrieval rates are generally greater with transthoracic approaches, resulting in superior TO rates.^20^ Our study reports that 93.9% of esophagectomy patients had at least 15 lymph nodes examined. Centers utilizing both approaches have reported this as the least achieved parameter.^3^^,^^10^ Using multivariable regression, Busweiler et al.^3^ even found that the transhiatal approach was a predictor of non-TO. Additionally, our center’s preferential use of neoadjuvant chemotherapy over chemoradiotherapy may explain our lymph node retrieval rates as chemoradiotherapy is known to reduce lymph node detection on postoperative histological specimens.^21^ Interestingly, the proportion of esophagectomy patients undergoing neoadjuvant therapy (78.7%) was lower than Dutch studies reporting rates between 90 and 100%.^3^^,^^7^^,^^8^ We cannot associate this with the lower rates of R0 resection in our cohort (70.6%) as these R0 rates are difficult to compare due to differing definitions of margin-negative resection. In the UK, R0 resection mandates that the carcinoma is >1 mm from the surgical resection margin,^11^ however, Dutch pathologists define it as no involvement of the resection margin (0 mm). The Dutch definition would incorporate R1a resections in UK centers as R0. Including R1a esophagectomy patients increases the R0 resection rate to 87.7% in our center. Hence, the stricter definition in the UK reduces our center’s R0 rate.

Importantly, discrepancy in TO rates cannot be entirely explained by center management alone: Van der Kaaij et al.^7^ report superior TO rates of 40.3%, utilizing neoadjuvant chemoradiotherapy in all esophagectomy patients and the transhiatal approach in nearly 69%. TO rates also correlate with increasing hospital case volume: an international audit reported rates of 36% in low-volume centers and 44% in centers performing more than 50 cases annually.^2^ Moreover, differences in study period lengths account for disparity in TO rates, as studies with earlier populations would be expected to have lower rates.^22^ As there is currently no validated universal consensus on the TO definition, its parameters still differ between studies, limiting comparability.^23^ The revision of parameters, including the reclassification of severe postoperative complications to those greater than Clavien–Dindo III, can overestimate TO rates.

Gastrectomy procedures are associated with lower postoperative morbidity in comparison to esophagectomy,^24^ which accounts for the higher TO rate reported in our study. In sum, 51.3% achieved TO, which is favorable compared to reported rates between 32.1 and 45.7% by centers that defined TO according to the DUCA.^3^^,^^7^^,^^10^^,^^24^ This could be attributed to the specialist and high-volume nature of our institution; however, we acknowledge that variation in TO rates is multifactorial.

Identifying predictors of TO can lead to quality improvement through the detection of high-risk patients. In gastrectomy patients, an increase in BMI by one unit corresponded to a 7% decrease in the likelihood of TO (OR: 0.93, 95% CI (0.88–0.98); *p* = 0.011). A high BMI had a similar effect in the esophagectomy cohort; however, this was not statistically significant (*p* = 0.360). The weaker effect in these patients may be attributed to the compensatory association of a low patient BMI and poor postoperative outcomes due to poor surgical tolerance.^25^ Meta-analysis evidence demonstrates that obesity increases the risk of complications following esophagectomy and gastrectomy.^25^^,^^26^ This is attributed to the prolongation of anesthesia and intubation, the effect of comorbidities such as diabetes, and poorer postoperative healing leading to higher anastomotic leak rates.^25^^,^^26^ Our findings implicate the value of prehabilitation to reduce BMI and improve TO rates following OGC surgery.^27^ Cho et al.^28^ evaluated a preoperative regimen consisting of aerobic exercise and resistance training in gastrectomy patients with metabolic syndrome and demonstrated a significant decrease in the BMI and postoperative morbidity in the exercise group. Although the evidence to mandate prehabilitation in OGC patients with a high BMI is limited, the implementation of a training program would at least serve to improve their quality of life, cancer-related fatigue, and physical functioning.^27^

T3 and T4 pathological tumor stages were identified as predictors of non-TO in the esophagectomy cohort. High tumor stage potentially complicates resection and increases morbidity.^14^ Neoadjuvant therapy is utilized for downstaging and improving the rate of R0 resection in patients with locally advanced disease.^29^ In accordance with guidelines, OGC patients at the QEHB are offered neoadjuvant chemotherapy; however, 60.6% of patients remained pT3–4 at surgery.^30^ The most optimal neoadjuvant chemotherapeutic regimen is disputed, and research into treatment cycles and the role of immunological therapies can augment the efficacy of neoadjuvant therapy, improving perioperative outcomes.^31^ National audit data show that 43% of OGC patients are diagnosed with stage IV disease, and this regularity of late-stage disease equally indicates the burden of late presentation.^32^ Besides symptomatic presentation in advanced stages, delays in seeking primary care prolong diagnosis, contributing to disease progression.^33^ Stage at diagnosis was not associated with socioeconomic disparities,^32^ but strategies to improve symptom awareness can reduce stage at diagnosis and improve outcomes.

Some studies identify minimally invasive approaches as predictors of TO,^2^^,^^10^^,^^22^ potentially due to reduced postoperative morbidity, shorter recovery time and improved lymph node yield in comparison to open approaches.^34^^,^^35^ This finding was not replicated in our study with laparoscopic gastrectomy instead favoring non-TO (OR: 0.35, CI [0.12–0.97]; *p* = 0.050). This association could be attributed to the technical complexity of these procedures, the low numbers performed in our unit, and potential learning curve. Brenkman et al.^36^ demonstrated that its centers required 51 laparoscopic gastrectomy cases before reaching a plateau in the TO rate.

We demonstrated significant association between TO attainment and survival. This is unsurprising considering TO contains several long-term prognostic factors, including R0 resection; however, this supports its use as a surrogate measure of beneficial long-term outcomes.^8^ TO provides a comprehensive assessment of the quality of perioperative care, enabling comparison of healthcare providers. As an all-or-nothing indicator, it sets a high standard and serves as an impetus to improve healthcare quality.^37^ Its utility is limited by its varying definition due to differences in data availability and lack of universally accepted consensus. A novel consensus-based definition for esophageal cancer patients, as proposed by Kalff et al.,^38^ amends debated parameters while increasing procedure-specificity with the inclusion of anastomotic leakage. Validating this definition and its applicability to gastrectomy patients can lead to standardization of TO in OGC surgery. This could facilitate objective comparisons of international specialist units and support the integration of TO into UK quality improvement analysis.

Our study has some inherent limitations with its single-center design as this limits generalizability. Nonetheless, the setting was a large tertiary hospital with a considerable caseload of upper gastrointestinal patients. We were unable to incorporate the Charlson Comorbidity Index into the multivariable model, which limited our ability to comprehensively account for comorbidities as a potential confounding factor. The inclusion of patients with postoperative mortality in the survival analysis will have overestimated the prognostic effect of TO attainment. However, we justify their inclusion to prevent the risk of survivorship bias.^9^

In conclusion, this study has demonstrated that our institution’s TO rates are concordant with other specialist centers. Public health measures to allow earlier diagnosis as well as research to optimize prehabilitation and patient BMI could improve perioperative outcomes in patients undergoing OGC surgery. Stepwise improvements in TO rates will potentially translate into superior long-term outcomes. TO can become a valuable metric in further quality improvement analyses; however, its integration is dependent on the establishment of a validated consensus-based definition.

## Supplementary Material

Supplementary_tables_revision_doae023

## Notes

Funding: We are grateful to the Arthur Thomson Trust and the Upper GI Patient support group for their funding.

Specific author contributions: Ganesh Velayudham (Conceptualization, Data curation, Formal analysis, Funding acquisition, Investigation, Methodology, Project administration, Validation, Writing—original draft, Writing—review & editing), Alexander Dermanis (Writing—original draft, Writing—review & editing), Sivesh Kamarajah (Conceptualization, Formal analysis, Supervision, Writing—review & editing), and Ewen Griffiths (Conceptualization, Project administration, Supervision, Writing—review & editing).

## References

[1] Cevasco M , Ashley S W. Quality measurement and improvement in general surgery. Perm J 2011; 15(4): 48–53. 10.7812/tpp/11-110 PMC3267561 22319416

[2] Kamarajah S K , Nepogodiev D, Hodson J et al. Textbook outcome following oesophagectomy for cancer: international cohort study. Br J Surg 2022; 109(5): 439–49. 35194634 10.1093/bjs/znac016

[3] Busweiler L A D , Schouwenburg M G, van Berge Henegouwen M I et al. Textbook outcome as a composite measure in oesophagogastric cancer surgery. Br J Surg 2017; 104(6): 742–50. 28240357 10.1002/bjs.10486

[4] Dimick J B , Welch H G, Birkmeyer J D. Surgical mortality as an indicator of hospital quality: the problem with small sample size. JAMA 2004; 292(7): 847–51. 15315999 10.1001/jama.292.7.847

[5] Lucocq J , Scollay J, Patil P. Evaluation of textbook outcome as a composite quality measure of elective laparoscopic cholecystectomy. JAMA Netw Open 2022; 5(9): E2232171. 36125810 10.1001/jamanetworkopen.2022.32171 PMC9490496

[6] Salet N , Bremmer R H, Verhagen M A M T et al. Is textbook outcome a valuable composite measure for short-term outcomes of gastrointestinal treatments in the Netherlands using hospital information system data? A retrospective cohort study. BMJ Open 2018; 8(2): e019405. 10.1136/bmjopen-2017-019405 PMC5855341 29496668

[7] van der Kaaij R T , de Rooij M V, van Coevorden F et al. Using textbook outcome as a measure of quality of care in oesophagogastric cancer surgery. Br J Surg 2018; 105(5): 561–9. 29465746 10.1002/bjs.10729

[8] Van Der Werf L R , Wijnhoven B P L, Fransen L F C et al. A national cohort study evaluating the association between short-term outcomes and long-term survival after esophageal and gastric cancer surgery. Ann Surg 2019; 270(5): 868–76. 31634182 10.1097/SLA.0000000000003520

[9] Kalff M C , Vesseur I, Eshuis W J et al. The association of textbook outcome and long-term survival after esophagectomy for esophageal cancer. Ann Thorac Surg 2021; 112(4): 1134–41. 33221197 10.1016/j.athoracsur.2020.09.035

[10] Bolger J C , Al Azzawi M, Whooley J et al. Surgery by a minimally invasive approach is associated with improved textbook outcomes in oesophageal and gastric cancer. Eur J Surg Oncol 2021; 47(9): 2332–9. 33766456 10.1016/j.ejso.2021.03.240

[11] Grabsch H , Mapstone N. Standards and datasets for reporting cancers. Dataset for histopathological reporting of oesophageal and gastric carcinoma Internet 2019; [cited 2019 October 01]. Available from: https://www.rcpath.org/static/f8b1ea3d-5529-4f85-984c8d4d8556e0b7/068e9093-0aea-4316-bdd49771564784b9/g006-dataset-for-histopathological-reporting-of-oesophageal-and-gastric-carcinoma.pdf.

[12] Dindo D , Demartines N, Clavien P A. Classification of surgical complications: a new proposal with evaluation in a cohort of 6336 patients and results of a survey. Ann Surg 2004; 240(2): 205–13. 15273542 10.1097/01.sla.0000133083.54934.ae PMC1360123

[13] Wu M Y , McGregor R J, Scott J et al. Textbook outcomes for oesophagectomy: a valid composite measure assessment tool for surgical performance in a specialist unit. Eur J Surg Oncol 2023; 49(9): 106897. 37032271 10.1016/j.ejso.2023.03.233

[14] Chen J Y , Lin G T, Chen Q Y et al. Textbook outcome, chemotherapy compliance, and prognosis after radical gastrectomy for gastric cancer: a large sample analysis. Eur J Surg Oncol 2022; 48(10): 2141–8. 35780034 10.1016/j.ejso.2022.05.025

[15] Sędłak K , Rawicz-Pruszyński K, Mlak R et al. Union is strength: textbook outcome with perioperative chemotherapy compliance decreases the risk of death in advanced gastric cancer patients. Eur J Surg Oncol 2022; 48(2): 356–61. 34404560 10.1016/j.ejso.2021.08.005

[16] Carbonell Morote S , Gracia Alegría E, de la Cuesta R et al. Textbook outcome in gastric surgery, what implications does it have on survival? Cir Esp English Edition 2023; 101(1): 20–8. 35787475 10.1016/j.cireng.2022.06.047

[17] Jamieson G G , Mathew G, Ludemann R, Wayman J, Myers J C, Devitt P G. Postoperative mortality following oesophagectomy and problems in reporting its rate. Br J Surg 2004; 91(8): 943–7. 15286953 10.1002/bjs.4596

[18] Charlson M E , Carrozzino D, Guidi J, Patierno C. Charlson Comorbidity Index: a critical review of clinimetric properties. Psychother Psychosom 2022; 91(1): 8–35. 34991091 10.1159/000521288

[19] Rice T W , Patil D T, Blackstone E H. 8th edition AJCC/UICC staging of cancers of the esophagus and esophagogastric junction: application to clinical practice. Ann Cardiothorac Surg 2017; 6(2): 119–30. 28447000 10.21037/acs.2017.03.14 PMC5387145

[20] Clemente-Gutiérrez U , Medina-Franco H, Santes O, Morales-Maza J, Alfaro-Goldaracena A, Heslin M J. Open surgical treatment for esophageal cancer: transhiatal vs transthoracic, does it really matter? J Gastrointest Oncol 2019; 10(4): 783–8. 31392059 10.21037/jgo.2019.03.12 PMC6657328

[21] Kauppila J H , Wahlin K, Lagergren P, Lagergren J. Neoadjuvant therapy in relation to lymphadenectomy and resection margins during surgery for oesophageal cancer. Sci Rep 2018; 8(1): 446. 29323261 10.1038/s41598-017-18879-6 PMC5765051

[22] Kulshrestha S , Bunn C, Patel P M et al. Textbook oncologic outcome is associated with increased overall survival after esophagectomy. Surgery 2020; 168(5): 953–61. 32675034 10.1016/j.surg.2020.05.038

[23] Ramia J M , Soria-Aledo V. Textbook outcome: a new quality tool. Cirugía Española (English Edition) 2022; 100(3): 113–4. 10.1016/j.cireng.2021.06.021 35216913

[24] Van Der Werf L R , Busweiler L A D, Van Sandick J W, Van Berge Henegouwen M I, Wijnhoven B P L. Reporting national outcomes after esophagectomy and gastrectomy according to the Esophageal Complications Consensus Group (ECCG). Ann Surg 2020; 271(6): 1095–101. 30676381 10.1097/SLA.0000000000003210

[25] Tsekrekos A , Lovece A, Chrysikos D et al. Impact of obesity on the outcomes after gastrectomy for gastric cancer: a meta-analysis. Asian J Surg 2022; 45(1): 15–26. 33965317 10.1016/j.asjsur.2021.04.033

[26] Gao H , Feng H M, Li B et al. Impact of high body mass index on surgical outcomes and long-term survival among patients undergoing esophagectomy: a meta-analysis. Medicine 2018; 97(28): e11091. 29995752 10.1097/MD.0000000000011091 PMC6076106

[27] Bausys A , Mazeikaite M, Bickaite K, Bausys B, Bausys R, Strupas K. The role of Prehabilitation in modern esophagogastric cancer surgery: a comprehensive review. Cancers (Basel) 2022; 14(9): 2096. 35565226 10.3390/cancers14092096 PMC9102916

[28] Cho H , Yoshikawa T, Oba M S et al. Matched pair analysis to examine the effects of a planned preoperative exercise program in early gastric cancer patients with metabolic syndrome to reduce operative risk: the Adjuvant Exercise for General Elective Surgery (AEGES) study group. Ann Surg Oncol 2014; 21(6): 2044–50. 24671637 10.1245/s10434-013-3394-7

[29] Ahn H S , Jeong S H, Son Y G et al. Effect of neoadjuvant chemotherapy on postoperative morbidity and mortality in patients with locally advanced gastric cancer. Br J Surg 2014; 101(12): 1560–5. 25200278 10.1002/bjs.9632

[30] NICE . Oesophago-gastric cancer: assessment and management in adults. NICE Internet 2018; [cited 2023 October 1]. Available from: https://www.nice.org.uk/guidance/ng83/chapter/Recommendations#assessment-after-diagnosis. 29400920

[31] Hou S , Pan Z, Hao X, Hang Q, Ding Y. Recent progress in the neoadjuvant treatment strategy for locally advanced esophageal cancer. Cancers (Basel) 2021; 13(20): 5162. 10.3390/cancers13205162 PMC8533976 34680311

[32] National Oesophago-Gastric Cancer Audit. 2022 Annual Report . [Internet]. 2023 [cited 2023 October 1]. Available from: https://www.nogca.org.uk/reports/2022-annual-report/

[33] van Erp N F, Helsper C W, Slottje P et al. Time to diagnosis of symptomatic gastric and oesophageal cancer in the Netherlands: where is the room for improvement? United Eur Gastroenterol J 2020; 8(5): 607–20. 10.1177/2050640620917804 PMC7268938 32250202

[34] Lordick F , Carneiro F, Cascinu S et al. Gastric cancer: ESMO clinical practice guideline for diagnosis, treatment and follow-up. Ann Oncol 2022; 33(10): 1005–20. 35914639 10.1016/j.annonc.2022.07.004

[35] Obermannová R , Alsina M, Cervantes A et al. Oesophageal cancer: ESMO clinical practice guideline for diagnosis, treatment and follow-up. Ann Oncol 2022; 33(10): 992–1004. 35914638 10.1016/j.annonc.2022.07.003

[36] Brenkman H J F , Claassen L, Hannink G et al. Learning curve of laparoscopic gastrectomy: a multicenter study. Ann Surg 2023; 277(4): E808–16. 35801714 10.1097/SLA.0000000000005479

[37] Nolan T , Berwick D M. All-or-none measurement raises the bar on performance. JAMA 2006; 295(10): 1168–70. 16522838 10.1001/jama.295.10.1168

[38] Kalff M C , Van Berge Henegouwen M I, Gisbertz S S. Textbook outcome for esophageal cancer surgery: an international consensus-based update of a quality measure. Dis Esophagus 2021; 34(7): 1–7. 10.1093/dote/doab011 PMC8275976 33744921

