# Supplementary material for: Predictors of textbook outcome following oesophagogastric cancer surgery
Source: Dis Esophagus. 2024 Mar 24;37(7):doae023. doi: 10.1093/dote/doae023 (PMC11220663; doi:10.1093/dote/doae023)
Supplement: Supplementary_tables_revision_doae023 [file supplementary_tables_revision_doae023.docx]

**Predictors of textbook outcome following oesophagogastric cancer surgery**

G. Velayudham, A. Dermanis, S.Kamarajah, E. Griffiths

**Supplementary tables**

**Table S1: Univariable and multivariable Cox model for overall survival in study patients with gastric cancer (n=312)**

|  |  | **Univariable** | **Multivariable** |  |
| --- | --- | --- | --- | --- |
| **Age** | - | **1.03 (1.01-1.04, p=0.001)** | 1.02 (1.00-1.04, p=0.029) |  |
| **Gender** | F | Ref- | Ref- |  |
|  |  |  |  |  |
|  | M | 0.85 (0.60-1.20, p=0.355) | 0.86 (0.58-1.27, p=0.451) |  |
| **BMI** | - | **0.96 (0.92-0.99, p=0.018)** | **0.94 (0.90-0.98, p=0.002)** |  |
| **ASA Grade** | 1 | Ref- | Ref- |  |
|  |  |  |  |  |
|  | 2 | 1.08 (0.56-2.10, p=0.823) | 0.95 (0.44-2.06, p=0.899) |  |
|  | 3 | **2.09 (1.08-4.05, p=0.030)** | 1.54 (0.70-3.39, p=0.288) |  |
|  | 4 | **4.66 (1.59-13.68, p=0.005)** | 3.13 (0.86-11.33, p=0.082) |  |
| **Comorbidity count** | 0 | Ref- | Ref- |  |
|  |  |  |  |  |
|  | 1 | 1.02 (0.67-1.56, p=0.923) | 0.99 (0.62-1.59, p=0.962) |  |
|  | >2 | 1.07 (0.73-1.58, p=0.714) | 1.03 (0.65-1.62, p=0.898) |  |
| **Tumour Histology** | Adenocarcinoma | Ref- | Ref- |  |
|  |  |  |  |  |
|  | Other | 0.69 (0.17-2.78, p=0.600) | 0.82 (0.17-4.01, p=0.809) |  |
| **T Stage** | T0 | Ref- | Ref- |  |
|  |  |  |  |  |
|  | T1 | 0.83 (0.24-2.83, p=0.768) | 0.77 (0.22-2.76, p=0.693) |  |
|  | T2 | 1.21 (0.36-4.04, p=0.759) | 1.28 (0.36-4.53, p=0.705) |  |
|  | T3 | 2.26 (0.71-7.25, p=0.168) | 1.54 (0.45-5.28, p=0.490) |  |
|  | T4 | **4.59 (1.44-14.68, p=0.010)** | 2.09 (0.60-7.35, p=0.250) |  |
| **N Stage** | N0 | Ref- | Ref- |  |
|  |  |  |  |  |
|  | N1 | **1.96 (1.23-3.11, p=0.004)** | 1.44 (0.85-2.45, p=0.179) |  |
|  | N2 | **3.45 (2.22-5.39, p<0.001)** | **2.73 (1.63-4.56, p<0.001)** |  |
|  | N3 | **5.63 (3.65-8.70, p<0.001)** | **2.87 (1.67-4.94, p<0.001)** |  |
| **M Stage** | M0 | Ref- | Ref- |  |
|  |  |  |  |  |
|  | M1 | **4.06 (1.97-8.37, p<0.001)** | **2.62 (1.12-6.11, p=0.026)** |  |
| **Neoadjuvant therapy** | No | Ref- | Ref- |  |
|  |  |  |  |  |
|  | Yes | 0.97 (0.71-1.33, p=0.856) | 1.01 (0.68-1.51, p=0.951) |  |
| **Surgical approach** | Open | Ref- | Ref- |  |
|  |  |  |  |  |
|  | Laparoscopic | 0.54 (0.29-1.01, p=0.053) | 0.77 (0.38-1.57, p=0.476) |  |
| **Anastomosis type** | Circular Stapled | Ref- | Ref- |  |
|  |  |  |  |  |
|  | Hand Sewn | 0.89 (0.59-1.34, p=0.574) | 1.00 (0.63-1.58, p=0.992) |  |
|  | Linear Stapled | 0.71 (0.48-1.06, p=0.097) | 0.60 (0.38-0.95, p=0.031) |  |
|  | Missing | **0.39 (0.21-0.73, p=0.003)** | 0.68 (0.33-1.41, p=0.303) |  |
| **Textbook outcome** | No | Ref- | Ref- |  |
|  |  |  |  |  |
|  | Yes | **0.62 (0.45-0.85, p=0.003)** | **0.63 (0.43-0.91, p=0.014)** |  |

Results reported as HR (95% CI, p-value). Dependent variable was death, hence, HR<1.0 favours OS. Significant results (p<0.05) are indicated in bold.

BMI, Body Mass Index; ASA, American Society of Anaesthesiologists

**Table S2: Univariable and multivariable Cox model for recurrence-free survival in study patients with gastric cancer (n=312)**

|  |  | **Univariable** | **Multivariable** |
| --- | --- | --- | --- |
| **Age** | - | **1.03 (1.01-1.04, p<0.001)** | **1.02 (1.00-1.04, p=0.016)** |
| **Gender** | F | Ref- | Ref- |
|  | M | 0.89 (0.63-1.24, p=0.485) | 0.90 (0.61-1.31, p=0.577) |
| **BMI** | - | **0.96 (0.93-0.99, p=0.017)** | **0.94 (0.90-0.98, p=0.002)** |
| **ASA Grade** | 1 | Ref- | Ref- |
|  | 2 | 1.07 (0.57-2.02, p=0.833) | 0.98 (0.47-2.04, p=0.949) |
|  | 3 | **1.90 (1.01-3.58, p=0.048)** | 1.23 (0.58-2.62, p=0.589) |
|  | 4 | **3.78 (1.31-10.90, p=0.014)** | 2.25 (0.65-7.84, p=0.203) |
| **Comorbidity count** | 0 | Ref- | Ref- |
|  | 1 | 0.98 (0.64-1.48, p=0.911) | 0.99 (0.62-1.58, p=0.959) |
|  | >2 | 1.09 (0.75-1.59, p=0.640) | 1.18 (0.75-1.84, p=0.481) |
| **Tumour Histology** | Adenocarcinoma | Ref- | Ref- |
|  | Other | 0.65 (0.16-2.61, p=0.539) | 0.74 (0.15-3.57, p=0.708) |
| **T Stage** | T0 | Ref- | Ref- |
|  | T1 | 0.79 (0.23-2.70, p=0.708) | 0.70 (0.20-2.50, p=0.587) |
|  | T2 | 1.34 (0.40-4.44, p=0.635) | 1.27 (0.36-4.46, p=0.709) |
|  | T3 | 2.50 (0.78-7.98, p=0.123) | 1.66 (0.49-5.64, p=0.420) |
|  | T4 | **4.75 (1.49-15.17, p=0.009)** | 2.30 (0.66-8.02, p=0.191) |
| **N Stage** | N0 | Ref- | Ref- |
|  | N1 | **1.86 (1.18-2.93, p=0.008)** | 1.30 (0.77-2.20, p=0.321) |
|  | N2 | **3.87 (2.52-5.94, p<0.001)** | **3.13 (1.89-5.18, p<0.001)** |
|  | N3 | **5.42 (3.55-8.29, p<0.001)** | **2.79 (1.64-4.74, p<0.001)** |
| **M stage** | M0 | Ref- | Ref- |
|  | M1 | **3.33 (1.62-6.85, p=0.001)** | 1.96 (0.86-4.49, p=0.112) |
| **Neoadjuvant therapy** | No | Ref- | Ref- |
|  | Yes | 0.98 (0.72-1.34, p=0.923) | 0.99 (0.67-1.47, p=0.964) |
| **Surgical Approach** | Open | Ref- | Ref- |
|  | Laparoscopic | **0.51 (0.27-0.95, p=0.034)** | 0.66 (0.33-1.32, p=0.238) |
| **Anastomosis type** | Circular Stapled | Ref- | Ref- |
|  | Hand Sewn | 0.90 (0.60-1.34, p=0.593) | 1.04 (0.67-1.62, p=0.860) |
|  | Linear Stapled | 0.71 (0.48-1.06, p=0.096) | 0.66 (0.42-1.04, p=0.072) |
|  | Missing | **0.41 (0.22-0.74, p=0.003)** | 0.78 (0.39-1.57, p=0.488) |
| **Textbook outcome** | No | Ref- | Ref- |
|  | Yes | **0.62 (0.46-0.85, p=0.003)** | **0.64 (0.44-0.91, p=0.013)** |

Results reported as HR (95% CI, p-value). Dependent variable was recurrence/mortality, hence, HR<1.0 favours RFS. Significant results (p<0.05) are indicated in bold.

BMI, Body Mass Index; ASA, American Society of Anaesthesiologists

**Table S3: Univariable and multivariable Cox model for overall survival in study patients with oesophageal cancer (n=667)**

|  |  | **Univariable** | **Multivariable** |  |
| --- | --- | --- | --- | --- |
| **Age** | - | **1.01 (1.00-1.03, p=0.008)** | **1.02 (1.01-1.03, p=0.006)** |  |
| **Gender** | F | Ref- | Ref- |  |
|  |  |  |  |  |
|  | M | 1.03 (0.81-1.31, p=0.798) | 1.19 (0.90-1.56, p=0.215) |  |
| **BMI** | - | 0.99 (0.97-1.01, p=0.260) | 0.99 (0.97-1.02, p=0.645) |  |
| **ASA Grade** | 1 | Ref- | Ref- |  |
|  |  |  |  |  |
|  | 2 | **0.68 (0.51-0.89, p=0.005)** | **0.71 (0.53-0.94, p=0.019)** |  |
|  | 3 | 1.01 (0.75-1.38, p=0.930) | 0.86 (0.62-1.19, p=0.356) |  |
|  | 4 | 0.74 (0.30-1.83, p=0.511) | 0.65 (0.26-1.67, p=0.372) |  |
|  | Missing | 0.50 (0.18-1.39, p=0.184) | 0.60 (0.20-1.84, p=0.373) |  |
| **Comorbidity count** | 0 | Ref- | Ref- |  |
|  |  |  |  |  |
|  | 1 | **1.40 (1.11-1.77, p=0.005)** | 1.25 (0.97-1.62, p=0.086) |  |
|  | >2 | 1.14 (0.90-1.45, p=0.274) | 1.02 (0.77-1.34, p=0.905) |  |
| **Tumour Histology** | Adenocarcinoma | Ref- | Ref- |  |
|  |  |  |  |  |
|  | Other | **1.98 (1.20-3.28, p=0.008)** | 1.24 (0.73-2.10, p=0.429) |  |
|  | SCC | 1.03 (0.78-1.35, p=0.857) | 1.16 (0.85-1.59, p=0.354) |  |
| **T Stage** | T0 | Ref- | Ref- |  |
|  |  |  |  |  |
|  | T1 | 0.95 (0.54-1.65, p=0.846) | 0.86 (0.48-1.55, p=0.622) |  |
|  | T2 | 1.14 (0.65-2.01, p=0.650) | 0.81 (0.45-1.46, p=0.489) |  |
|  | T3 | **2.18 (1.34-3.57, p=0.002)** | 1.19 (0.71-2.01, p=0.508) |  |
|  | T4 | **4.40 (2.43-7.96, p<0.001)** | **2.55 (1.35-4.82, p=0.004)** |  |
| **N Stage** | N0 | Ref- | Ref- |  |
|  |  |  |  |  |
|  | N1 | **2.20 (1.75-2.77, p<0.001)** | **1.86 (1.44-2.41, p<0.001)** |  |
|  | N2 | **2.74 (2.02-3.71, p<0.001)** | **2.04 (1.46-2.86, p<0.001)** |  |
|  | N3 | **4.08 (2.90-5.74, p<0.001)** | **3.27 (2.23-4.81, p<0.001)** |  |
| **M Stage** | M0 | Ref- | Ref- |  |
|  |  |  |  |  |
|  | M1 | **2.34 (1.16-4.71, p=0.018)** | 1.97 (0.91-4.24, p=0.083) |  |
| **Neoadjuvant therapy** | No | Ref- | Ref- |  |
|  |  |  |  |  |
|  | Yes | 1.26 (0.99-1.61, p=0.061) | 1.06 (0.79-1.41, p=0.698) |  |
| **Surgical Approach** | Open | Ref- | Ref- |  |
|  |  |  |  |  |
|  | Hybrid | 0.91 (0.72-1.15, p=0.430) | 0.93 (0.70-1.23, p=0.608) |  |
|  | Total MIO | 0.78 (0.58-1.05, p=0.105) | 0.75 (0.56-1.03, p=0.072) |  |
| **Anastomosis type** | Circular Stapled | Ref- | Ref- |  |
|  |  |  |  |  |
|  | Hand Sewn | **1.28 (1.01-1.62, p=0.038)** | 1.22 (0.92-1.61, p=0.160) |  |
|  | Linear Stapled | 1.12 (0.87-1.46, p=0.379) | 1.30 (0.95-1.77, p=0.097) |  |
|  | Missing | **1.66 (1.07-2.59, p=0.023)** | 1.13 (0.70-1.83, p=0.616) |  |
| **Textbook outcome** | No | Ref- | Ref- |  |
|  |  |  |  |  |
|  | Yes | **0.54 (0.43-0.67, p<0.001)** | **0.60 (0.48-0.76, p<0.001)** |  |

Results reported as HR (95% CI, p-value). Dependent variable was death, hence, HR<1.0 favours OS. Significant results (p<0.05) are indicated in bold.

BMI, Body Mass Index; ASA, American Society of Anaesthesiologists; SCC, squamous cell carcinoma; MIO, minimally invasive oesophagectomy

**Table S4: Univariable and multivariable Cox model for recurrence-free survival in study patients with oesophageal cancer (n=667)**

|  |  | **Univariable** | **Multivariable** |
| --- | --- | --- | --- |
| **Age** | - | 1.01 (1.00-1.02, p=0.167) | 1.01 (1.00-1.02, p=0.104) |
| **Gender** | F | Ref- | Ref- |
|  | M | 1.01 (0.80-1.28, p=0.940) | 1.16 (0.89-1.52, p=0.270) |
| **BMI** | - | 0.99 (0.97-1.01, p=0.342) | 1.00 (0.98-1.02, p=0.838) |
| **ASA Grade** | 1 | Ref- | Ref- |
|  | 2 | **0.70 (0.53-0.92, p=0.011)** | 0.73 (0.55-0.98, p=0.037) |
|  | 3 | 1.00 (0.73-1.35, p=0.975) | 0.85 (0.61-1.17, p=0.315) |
|  | 4 | 0.68 (0.27-1.70, p=0.411) | 0.57 (0.22-1.46, p=0.242) |
|  | Missing | 0.53 (0.19-1.48, p=0.227) | 0.60 (0.20-1.83, p=0.372) |
| **Comorbidity count** | 0 | Ref- | Ref- |
|  | 1 | **1.37 (1.09-1.73, p=0.007)** | **1.29 (1.00-1.66, p=0.046)** |
|  | >2 | 1.12 (0.89-1.42, p=0.329) | 1.08 (0.82-1.41, p=0.587) |
| **Tumour Histology** | Adenocarcinoma | Ref- | Ref- |
|  | Other | **1.94 (1.18-3.21, p=0.010)** | 1.23 (0.73-2.08, p=0.442) |
|  | SCC | 1.03 (0.78-1.35, p=0.834) | 1.17 (0.86-1.60, p=0.309) |
| **T Stage** | T0 | Ref- | Ref- |
|  | T1 | 0.96 (0.56-1.66, p=0.886) | 0.93 (0.53-1.65, p=0.811) |
|  | T2 | 1.22 (0.70-2.11, p=0.480) | 0.92 (0.52-1.62, p=0.779) |
|  | T3 | **2.40 (1.49-3.87, p<0.001)** | 1.39 (0.84-2.30, p=0.202) |
|  | T4 | **4.91 (2.75-8.76, p<0.001)** | **2.91 (1.57-5.43, p=0.001)** |
| **N Stage** | N0 | Ref- | Ref- |
|  | N1 | **2.23 (1.78-2.79, p<0.001)** | **1.83 (1.43-2.36, p<0.001)** |
|  | N2 | **2.91 (2.17-3.91, p<0.001)** | **2.11 (1.52-2.91, p<0.001)** |
|  | N3 | **4.32 (3.10-6.01, p<0.001)** | **3.24 (2.24-4.70, p<0.001)** |
| **M stage** | M0 | Ref- | Ref- |
|  | M1 | **2.29 (1.13-4.60, p=0.021)** | 1.72 (0.80-3.69, p=0.161) |
| **Neoadjuvant therapy** | No | Ref- | Ref- |
|  | Yes | **1.37 (1.08-1.75, p=0.010)** | 1.07 (0.80-1.43, p=0.635) |
| **Surgical Approach** | Open | Ref- | Ref- |
|  | Hybrid | 0.85 (0.67-1.07, p=0.166) | 0.85 (0.65-1.11, p=0.222) |
|  | Laparoscopic | 0.78 (0.59-1.04, p=0.090) | 0.74 (0.55-1.00, p=0.053) |
| **Anastomosis type** | Circular Stapled | Ref- | Ref- |
|  | Hand Sewn | **1.31 (1.04-1.65, p=0.024)** | 1.30 (0.99-1.70, p=0.059) |
|  | Linear Stapled | 1.08 (0.84-1.39, p=0.546) | 1.28 (0.95-1.73, p=0.105) |
|  | Missing | **1.64 (1.06-2.54, p=0.027)** | 1.18 (0.73-1.90, p=0.499) |
| **Textbook outcome** | No | Ref- | Ref- |
|  | Yes | **0.56 (0.46-0.70, p<0.001)** | **0.63 (0.51-0.79, p<0.001)** |

Results reported as HR (95% CI, p-value). Dependent variable was recurrence/mortality, hence, HR<1.0 favours RFS. Significant results (p<0.05) are indicated in bold.

BMI, Body Mass Index; ASA, American Society of Anaesthesiologists; SCC, squamous cell carcinoma; MIO, minimally invasive oesophagectomy
